# Deep Anterior Lamellar Keratoplasty on a Descemet′s Membrane Endothelial Keratoplasty Graft: A Case Report

**DOI:** 10.1155/crop/5652228

**Published:** 2026-05-14

**Authors:** Yuto Yukari, Takahiko Hayashi, Satoru Yamagami

**Affiliations:** ^1^ Department of Ophthalmology, Nihon University Itabashi Hospital, Tokyo, Japan, nihon-u.ac.jp

**Keywords:** *corneal irregular astigmatism*, deep anterior lamellar keratoplasty, Descemet′s membrane endothelial keratoplasty, therapeutic keratoplasty

## Abstract

**Introduction:**

Deep anterior lamellar keratoplasty (DALK) has been reported in eyes following Descemet′s stripping automated endothelial keratoplasty (DSAEK); however, its application after Descemet′s membrane endothelial keratoplasty (DMEK) has not been reported.

**Case Presentation:**

The patient was an 87‐year‐old woman who underwent repeat DMEK for late DMEK graft failure that had healed with corneal haze due to long‐term endothelial dysfunction. DALK was then performed to remove opacity and improve corneal irregular astigmatism. The corneal epithelium and stroma were removed via manual dissection to the pre‐Descemet′s layer. *Candida parapsilosis* was detected in a culture test of the obtained host corneal specimen. Because a fungal infection was detected, 0.1% voriconazole eye drops were administered in addition to antibiotic eye drops. Corneal clarity improved, but optical coherence tomography revealed cystoid macular edema (CME). Subtenon triamcinolone acetonide (STTA) injection was performed. The CME resolved 1 week after STTA injection, and the best corrected visual acuity was 0.5 (decimal) 1 month after STTA injection.

**Discussion:**

This case demonstrates that DALK can be safely performed after DMEK to treat stromal opacity and irregular astigmatism, with favorable visual and anatomical outcomes. Therapeutic DALK and prophylactic antifungal eye drops effectively treated the fungal infection and recurrence was not observed. This case highlights the clinical feasibility of staged keratoplasty and suggests that DALK may be considered a viable option for managing stromal opacity even after endothelial keratoplasty. These findings may contribute to expanding the surgical indications of DALK in complex corneal cases following endothelial keratoplasty.

## 1. Introduction

In recent years, the trend in preferred keratoplasty method has shifted from penetrating keratoplasty (PKP) to partial thickness keratoplasty, which avoids removal of the healthy corneal layer. For corneal stromal disorders, deep anterior lamellar keratoplasty (DALK) is one of the best surgical treatment options. For corneal endothelial disorders, Descemet′s stripping automated endothelial keratoplasty (DSAEK) or Descemet′s membrane endothelial keratoplasty (DMEK) can be used.

There are some advantages associated with performing partial thickness transplantation. For example, the rates of allograft rejection tend to be lower than those associated with PKP, and closed eye surgery may facilitate the avoidance of expulsive hemorrhage. Although the standard procedure for endothelial dysfunction is an endothelial keratoplasty operation such as DSAEK or DMEK, PKP may be used in cases with corneal stromal opacity or an irregular anterior corneal surface. Notably, there have been some reports of DALK performed in eyes that have undergone DSAEK [1], but no reports of DALK performed in eyes that have undergone DMEK. Herein, we describe a successful DALK procedure in a patient with corneal stromal opacity and corneal irregular astigmatism.

## 2. Case Report

This case report was prepared in accordance with the CARE guidelines. An 87‐year‐old woman was referred for the surgical treatment of cataracts and reduced endothelial cell density (ECD). In the right eye, the best corrected visual acuity (BCVA) was 0.4 (decimal), and the ECD was uncountable. Slit‐lamp biomicroscopy revealed guttae, and she was diagnosed with Fuchs endothelial corneal dystrophy. There was no clinically apparent corneal edema on slit‐lamp examination. The diameter of confluence was > 5 mm, and the modified Krachmer grading scale score was 5. Both eyes underwent phacoemulsification and intraocular lens implantation (PEA + IOL). The cornea of the right eye was clear, but the BCVA in that eye was 0.4 (decimal), and no improvement was observed. The right eye underwent DMEK 4 months after PEA + IOL to improve visual acuity.

**Figure 1:**
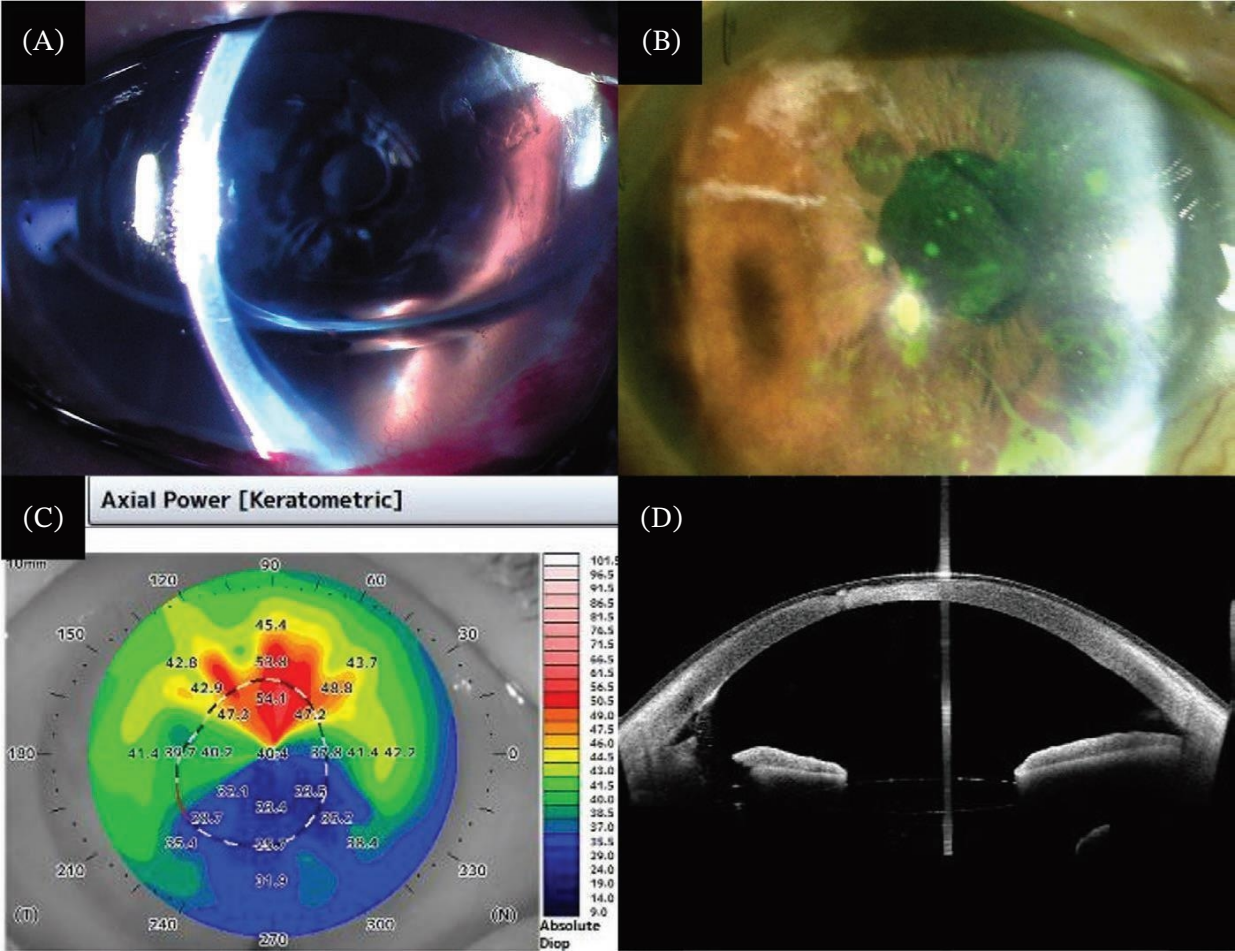
(A) Photograph of slit‐lamp biomicroscopy after repeat Descemet′s membrane endothelial keratoplasty (DMEK). The anterior chamber is filled with SF6 gas. (B) Photograph of slit‐lamp biomicroscopy 4 months after repeat DMEK. Corneal opacity was observed at the ulcerated location. (C, D) Anterior segment optical coherence tomography revealed an irregular astigmatism and corneal ulceration.

The patient underwent three rebubbling procedures for graft reattachment. The graft detachment resolved 2 months after surgery, but the corneal edema remained, and the BCVA was 0.1 (decimal). The patient was diagnosed with late endothelial failure, and repeat DMEK with a diameter of 8.0 mm was performed 6 months after the first DMEK (Figure 1A). After repeat DMEK, the corneal edema resolved to 392 *μ*m, but the BCVA was 0.04 (decimal). Postoperatively, the patient was treated with 0.1% betamethasone sodium phosphate (Santen) and 0.5% levofloxacin (Santen), administered four times daily and 0.1% bromfenac sodium (Senju) administered two times daily. No bacteria were detected in culture tests of the donor cornea or its preservation solution. The BCVA did not improve further due to corneal thinning related to dry eye and corneal irregular astigmatism. The refraction was sphere +4.00 D and cylinder −2.00‐D axis 170°. The topographic astigmatism measured by anterior segment optical coherence tomography (AS‐OCT) showed a high corneal astigmatism (7.1 D). Topical bromfenac was discontinued to improve the corneal epithelial disorder. A circle‐shaped corneal ulcer appeared 2 months after the repeat DMEK. Topical betamethasone was discontinued due to the possibility of corneal epithelial infection, but corneal opacity with ulceration appeared 4 months after the repeat DMEK (Figure 1B−D).

**Figure 2:**
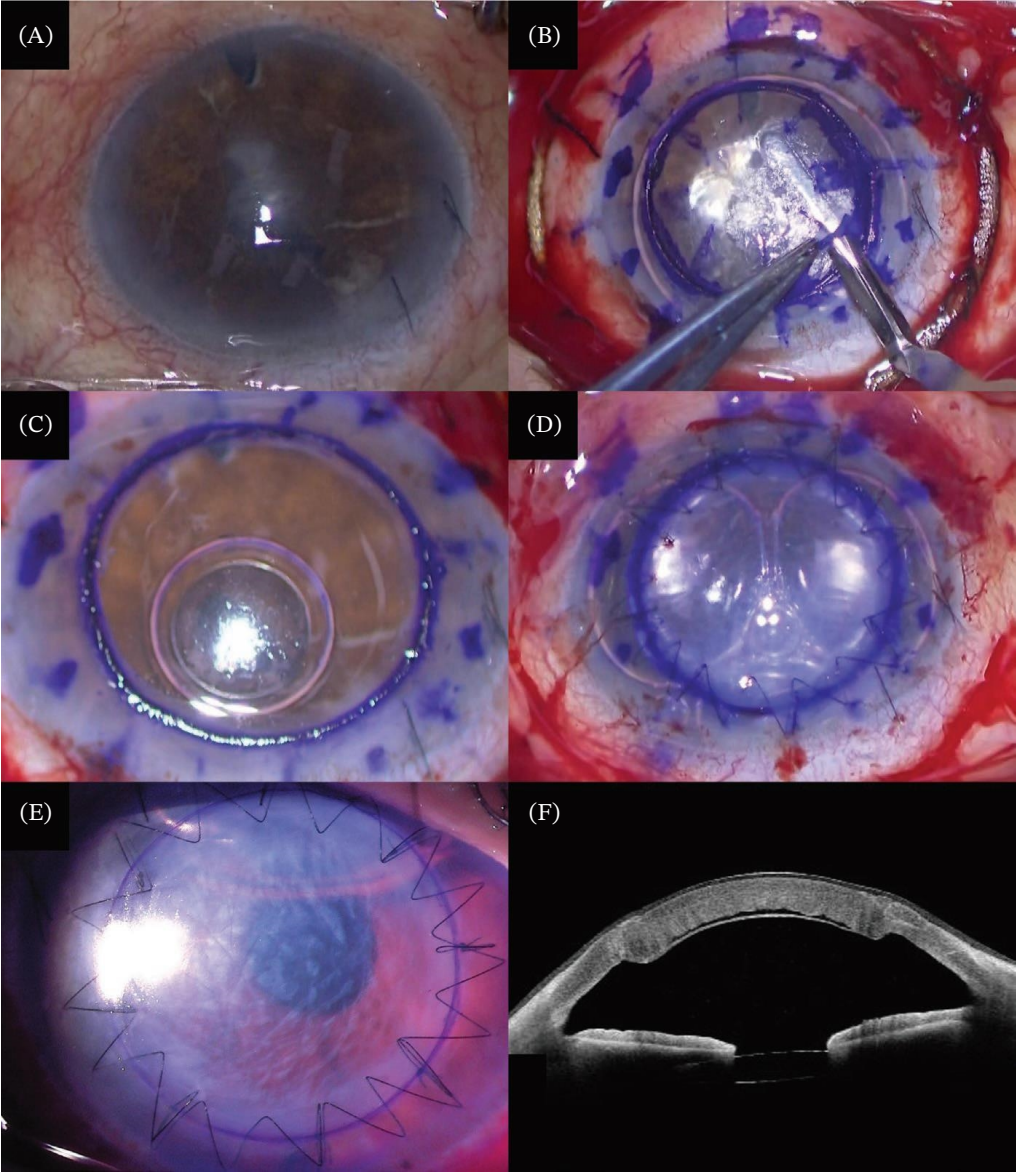
(A–D) Surgical steps of deep anterior lamellar keratoplasty (DALK) in an eye that has undergone Descemet′s membrane endothelial keratoplasty (DMEK). (B, C) The corneal epithelium and stroma were removed using manual dissection to the pre‐Descemet′s layer. (E) Photograph of slit‐lamp biomicroscopy 1 day after DALK. (F) Anterior segment optical coherence tomography revealed a minor space between each graft 1 day after DALK, which was absorbed within 2 weeks.

DALK was performed to remove the opacity and improve the corneal irregular astigmatism (Figure 2A). A large 7‐mm perforation from the center of the cornea was created using a corneal trephine (Barron Radial Vacuum Trephine, Corza Medical, United States). The corneal epithelium and stroma were removed via manual dissection (layer‐by‐layer technique) to the pre‐Descemet′s layer (Dua′s layer) (Figure 2B,C). Intraoperative optical coherence tomography (OCT) was used to confirm residual stromal thickness. The donor cornea was punched out at a diameter of 7.5 mm using a cornea punch (Barron Vacuum Donor Cornea Punch, Corza Medical, United States), and donor Descemet′s stripping was performed. The graft was secured with eight 10–0 nylon interrupting sutures (10–0 Nylon, Mani, Tochigi), then running sutured with sixteen 10–0 nylon stitches (Figure 2C,D). A space was observed between each graft for several days postoperatively (Figure 2E,F).

**Figure 3:**
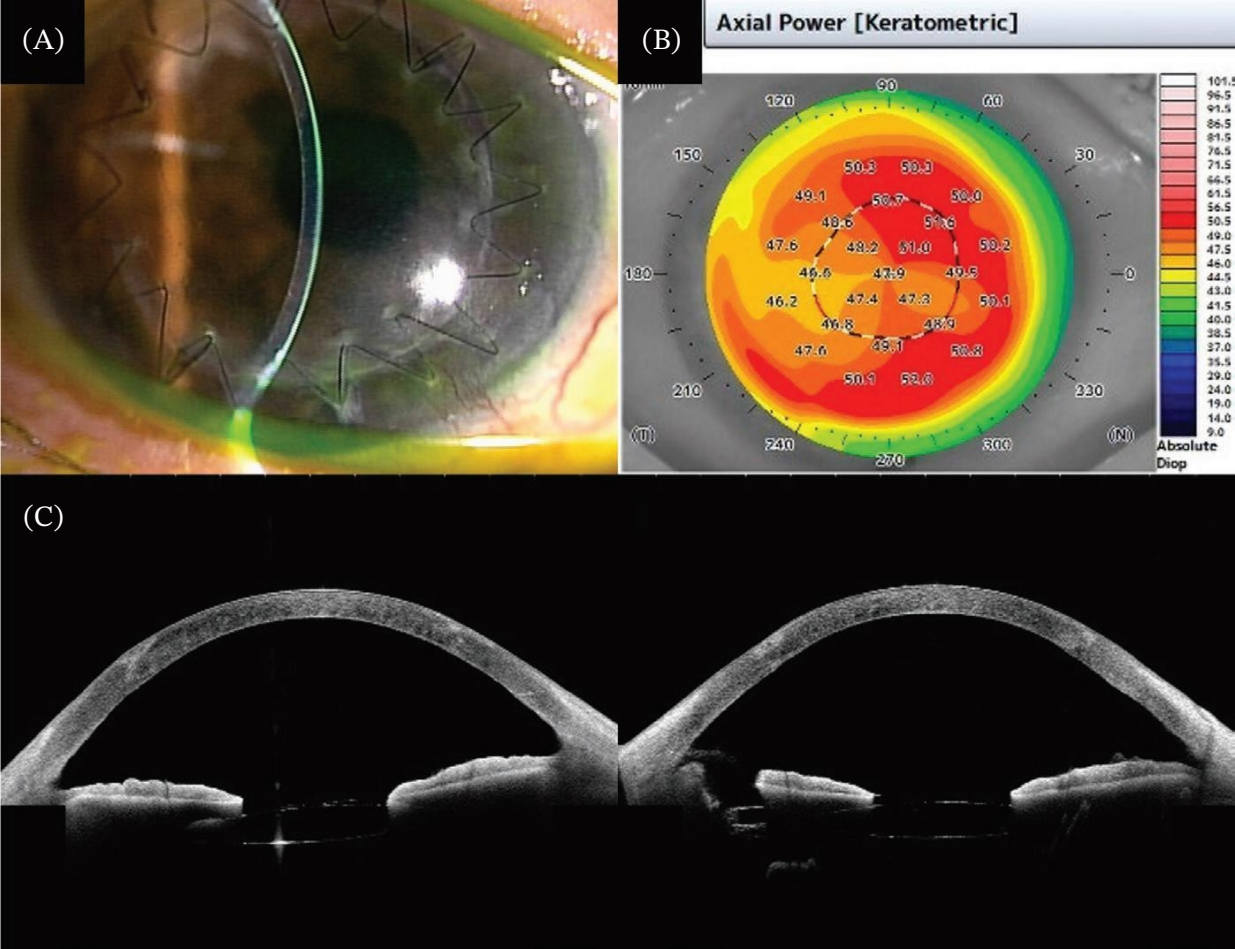
(A) Photograph of slit‐lamp biomicroscopy 6 months after deep anterior lamellar keratoplasty (DALK). (B, C) Anterior segment optical coherence tomography revealed improvement of the corneal irregular astigmatism 6 months after DALK.

Postoperatively, the patient was treated with 0.5% levofloxacin and 0.1% fluorometholone (Santen) administered four times daily. *Candida parapsilosis* and *Staphylococcus epidermidis* were detected in a culture test of the host cornea that had been removed. Because a fungal infection was detected, topical fluorometholone was discontinued, and 0.1% voriconazole was administered four times daily. The corneal clarity and corneal irregular astigmatism both improved (Figure 3). However, the BCVA remained poor at 0.3 (decimal) 5 months after DALK. Because OCT revealed cystoid macular edema (CME), a subtenon triamcinolone acetonide (STTA) injection was administered. The CME resolved 1 week postinjection, and the BCVA was 0.5 (decimal) 1 month postinjection. The postoperative refraction was sphere −3.00 D and cylinder −2.50‐D axis 160°. The topographic astigmatism measured by AS‐OCT showed a significant reduction in astigmatism from 7.1 to 2.2 D. As this is a single case report, no statistical analyses were performed.

## 3. Discussion

To our knowledge, this is the first report describing DALK performed on an eye that had previously undergone DMEK. Although DALK after DSAEK has been documented, the significantly thinner graft of DMEK presents unique intraoperative risks and considerations.

The main aspects of the above‐described case were as follows:

1. The indication for DALK in an eye that had undergone DMEK and its advantages.
2. The surgical technique used for DALK in an eye that had undergone DMEK.
3. The effects of the therapeutic DALK procedure and the risk of recurrence.
4. The difficulty of controlling inflammation after DALK in an eye that had undergone DMEK in an infected state.

First, we can consider the situations in which DALK is applicable after DMEK, and the advantages of performing the procedure. In the current case, dry eye was observed after DMEK, and corneal epithelial damage caused an irregular astigmatism. Culturing of the secondary DMEK donor tissue and preservation fluid revealed no fungal infection, and opacity occurred in an ulcerated area. These findings suggest that infection occurred during the postoperative period. Topical steroid eye drops were used to control rejection, and they may have induced the fungal infection. Therapeutic DALK was then performed to eliminate opacity and correct the irregular astigmatism.

If corneal stromal opacity or haze occurs after corneal endothelial transplantation, the surgical treatment options may include phototherapeutic keratectomy (PTK), PKP, and DALK [1]. Because PTK can only eliminate scars affecting superficial stromal thickness, it is not indicated for corneal opacity that is suspected to be located deeper in the corneal stroma, as in the current case. PKP entails a higher risk of graft failure and complications such as expulsive hemorrhage than DALK [2].

The surgical technique of chimeric transplantation procedures warrants discussion. There have been reports of DALK being performed on eyes that have undergone DSAEK [1], but there are no reports of DALK performed after DMEK. Kara [2] reported that when DALK and DMEK were both performed, DALK was performed first. The difference between DSAEK and DMEK lies in the graft thickness, as a DMEK graft is markedly thinner than a DSAEK graft. Therefore, when performing DALK in an eye that has undergone DMEK, greater care is required to avoid perforation. In the current case, the DMEK graft was not exposed, but Dua′s layer of the host cornea was exposed.

We must consider the safest method for preventing recurrence after therapeutic keratoplasty and the regimen of medical treatment. In cases of dry eye syndrome with chronic ulceration, the abnormal cornea is commonly infected with *Candida*. In particular, *C. parapsilosis* is found with increasing frequency [3, 4]. In a study investigating *C. parapsilosis* infection after corneal transplantation, therapeutic PKP was performed in some cases for debridement purposes. Topical voriconazole has also been used to prevent postoperative relapse [4], and Sarnicola et al. [5] described a safe method of performing therapeutic DALK for infectious keratitis such as fungal keratitis or acanthamoeba keratitis. One concern with therapeutic DALK is the recurrence of infection. The recurrence rate of infection in therapeutic DALK is reportedly the same as that for PKP. In addition, performing therapeutic DALK earlier is effective with respect to removing the infected lesion [5].

If the dose of anti‐inflammatory eye drops administered is insufficient, side effects such as allograft rejection or CME may ensue.

In this case, CME was detected after DALK; however, the DMEK graft remained in situ, and the patient had undergone prior endothelial keratoplasty. CME can occur even long after DMEK, and endothelial keratoplasty is associated with a persistent risk of postoperative CME. In addition, multiple intraocular procedures, including repeated DMEK and subsequent DALK, may have resulted in cumulative surgical stress. Therefore, it is difficult to attribute CME to a single procedure. Previous studies have reported that CME occurs in approximately 13.8% of patients after DMEK [6].

With appropriate treatment, the BCVA is comparable to that in non‐CME cases [6]. In the current case, OCT revealed CME, and the BCVA improved after STTA.

In conclusion, although additional cases are needed, this report demonstrates that therapeutic DALK after DMEK followed by prophylactic antifungal eye drops was effective with a careful surgical technique.

## Author Contributions

Yuto Yukari was a clinical investigator and contributed to data collection, mainly writing this paper. Satoru Yamagami was a participating investigator and critically reviewed the study proposal. Takahiko Hayashi was a clinical investigator and critically reviewed the research content. All authors reviewed the manuscript.

## Funding

No funding was received for this manuscript.

## Disclosure

All authors have read and approved the final version of the manuscript. Takahiko Hayashi had full access to all of the data in this study and takes complete responsibility for the integrity of the data and the accuracy of the data analysis. Takahiko Hayashi affirms that this manuscript is an honest, accurate, and transparent account of the study being reported; that no important aspects have been omitted; and that any discrepancies have been explained.

## Ethics Statement

This retrospective case report was approved by the Ethical Review Board of Nihon University School of Medicine (Approval No. RK‐210807‐1). This study adhered to the tenets of the Declaration of Helsinki. Written informed consent for publication was obtained from the patient.

## Conflicts of Interest

The authors declare no conflicts of interest.

## Data Availability

Data are available upon reasonable request.
